# Fluopyram or Resistant Cultivars Manage *Meloidogyne arenaria* Infestation in Virginia-Type Peanut Production

**DOI:** 10.2478/jofnem-2025-0010

**Published:** 2025-04-22

**Authors:** Zane J. Grabau, Sabina Budhathoki, Rebeca Sandoval Ruiz, Chang Liu

**Affiliations:** Entomology and Nematology Department, Institute of Food and Agricultural Sciences, University of Florida Institute, 1881 Natural Area Drive, Gainesville, FL 32611, United States; Laboratory of Nematology, Crop Protection Research Center, Agronomy School, University of Costa Rica, San José, Costa Rica 2060; Department of Biochemistry, Molecular Biology, Entomology, & Plant Pathology, Mississippi State University, 46 Old Bully Blvd, Mississippi State, MS 39762

**Keywords:** *Arachis hypogea*, fluopyram, free-living nematodes, management, *Meloidogyne arenaria*, nematicide, peanut, resistance

## Abstract

*Meloidogyne arenaria* (peanut root-knot nematode, PRKN) is an important pest in peanut (*Arachis hypogea*) production in the United States, including specialty Virginia-type peanuts. Cultivars resistant to PRKN and nematicide application are two available methods for managing PRKN. The objectives of this study were to determine the impacts of resistant Virginia-type peanut cultivars (Georgia-19HP and TifJumbo) on (1) management of PRKN abundances and damage and (2) total free-living nematode soil abundances. A common susceptible cultivar (Bailey II) with or without in-furrow fluopyram nematicide was compared to the resistant cultivars without nematicide in field trials in Florida (2022 and 2023). Resistant cultivars reduced midseason PRKN abundances from roots by 92–98% and final PRKN soil abundances by 81–93% relative to the untreated susceptible cultivar. Fluopyram reduced midseason PRKN root abundances by 65–74% and final PRKN soil abundances by 42–51% relative to untreated susceptible. Although PRKN reproduced on peanuts, no damage symptoms were observed, yield did not vary by treatment in 2022, and yield was significantly greater for fluopyram than either resistant cultivar in 2023. Impacts on total free-living nematode soil abundances were inconsistent. In summary, either fluopyram or resistant cultivars are effective tools for managing PRKN abundances in Virginia-type peanuts.

Peanut (*Arachis hypogea* ssp. *hypogaea* var. *hypogaea*) is an important crop in the United States, with over 590,000 hectares planted and an estimated crop value of $1.47 billion (United States Dollars) in 2022 (USDA-NASS, 2023). United States peanut production is centered in the southern states, particularly the Southeast (USDA-NASS, 2023). Peanuts can be further categorized by “types” that have been selected and bred primarily for kernel parameters or other agronomic characteristics based on the market. Runner-type peanuts are the most commonly grown type and have medium-sized, uniform kernels that are used primarily for peanut butter production and confections (Wright et al., 2021). Other peanut types are grown on smaller acreage, often in specific geographic regions. Virginia-type peanuts are generally more common in Virginia, the Carolinas, and the Southwest but are also important in certain Florida locales (Wright et al., 2021). Virginiatype peanuts have large kernels selected for flavor and are often sold as roasted gourmet peanuts either in-shell or shelled, so they typically have a greater value than Runner-type peanuts (Wright et al., 2021).

*Meloidogyne arenaria* race 1 (peanut root-knot nematode, PRKN) is a major pest in peanut production, causing substantial damage (Holbrook et al., 2023; Grabau et al., 2024) as illustrated by a reported damage threshold of 1 egg/100 cm^3^ soil (McSorley et al., 1992). The main strategies for PRKN management include crop rotation, nematicide application, and the use of resistant cultivars. The use of resistant cultivars is a particularly attractive strategy because it provides more flexibility than crop rotation and may have lower cost and less risk to handlers and the environment than nematicides (Desaeger et al., 2020). In peanuts, highly resistant cultivars have been developed as a tool to combat PRKN (Simpson and Starr, 2001; Holbrook et al., 2008; Branch and Brenneman, 2020). Currently, PRKN resistance in commercial peanuts is derived from the cultivar COAN, which was the result of introgression PRKN resistance from *A. cardenasii* into Runner-type peanuts in a complex breeding effort (Simpson and Starr, 2001). Since that time, PRKN resistance from COAN has been incorporated into Runner-type cultivars such as Tifguard, TifNV-HighO/L, TifNV-HG, and Georgia-14N with an aim to improve yield, quality, and disease resistance compared with earlier PRKN-resistant cultivars (Holbrook et al., 2008; Holbrook et al., 2017; Branch and Brenneman, 2020; Holbrook et al., 2023).

Evidence suggests that a major resistance gene, variously called *Rma* (Nagy et al., 2010) or *Ma-1* (Burow et al., 2014), is involved in PRKN resistance inherited from COAN (Choi et al., 1999; Burow et al., 2014; Clevenger et al., 2017). Marker-assisted selection is commonly used (e.g., SCAR 197/909 and SSR-GM565) when developing PRKN-resistant peanut cultivars (Holbrook et al., 2017; Branch and Brenneman, 2023). Phenotypic validation is needed, though, because some cultivars designated as susceptible based on the common markers SCAR 197/909 and SSR-GM565 have been resistant in greenhouse or field testing, such as Georgia-14N (Branch et al., 2014; Branch and Brenneman, 2015). Hypotheses about this discrepancy include the involvement of multiple genes in COAN-derived resistance or marker inaccuracy due to recombination events in the gene region between *Rma* and markers (Branch et al., 2014). In either case, this emphasizes the need for field validation of resistant peanut cultivars.

Runner-type peanut cultivars that have the COAN resistance background successfully incorporated are highly resistant to PRKN in field production, with very little reproduction occurring based on final soil nematode counts (Holbrook et al., 2008; Holbrook et al., 2017) and PRKN abundances on roots during the season (Grabau et al., 2024). Furthermore, resistant cultivars provide excellent protection from PRKN damage, greatly increasing yield relative to susceptible cultivars when PRKN infestation is severe (Holbrook et al., 2008, 2017). For example, under PRKN pressure, the resistant cultivar Tifguard increased yield by an average of 80% relative to a common susceptible Runner-type cultivar at the time of field testing, Georgia Green (Holbrook et al., 2008). TifNV-High O/L resistant cultivar increased yield by 58% in a Georgia field study and 39–125% relative to a current common susceptible cultivar, Georgia-06G, in a Florida field study with severe PRKN pressure (Holbrook et al., 2017; Grabau et al., 2024).

Until recently, PRKN resistance had only been available in Runner-type peanut types, not specialty types such as Virginia-type, leaving specialty peanut producers with fewer management options. Because of their specialized market, some Virginiatype peanut growers employ peanut monoculture, which further limits their PRKN management options. Thus, the recent introduction of Virginia-type peanut cultivars with COAN-derived resistance to PRKN, Georgia-19HP (GA-19HP), and TifJumbo could be an important tool for growers (Branch and Brenneman, 2020; Holbrook et al., 2024). In registration release documentation, GA-19HP was reported to have a high level of resistance to PRKN based on molecular markers and phenotypic data, but neither PRKN counts nor galling data were provided due to the condensed format of cultivar registrations (Branch and Brenneman, 2020). TifJumbo produced no egg masses and few galls in greenhouse testing and consistently yielded better than the PRKN-susceptible Virgnia-type cultivar Bailey in field testing under severe PRKN pressure (Holbrook et al., 2024). However, neither PRKN population dynamic nor symptom (galling) data were provided with field testing of TifJumbo (Holbrook et al., 2024). Therefore, additional field testing of these Virginia-type peanut cultivars under PRKN pressure is needed to help guide management choices.

Furthermore, selecting either a susceptible cultivar with nematicide application or a resistant cultivar is a common choice when managing PRKN in peanuts, and more field research to guide this decision is needed. Fluopyram is a nematicide commonly used in United States peanut production. Fluopyram is a fluorinated nematicide, a new group of nematicides that generally have reduced risk to handlers and the environment relative to older insecticide-nematicides (Desaeger et al., 2020). Broadly across crops, fluopyram can help manage root-knot nematodes (Dahlin et al., 2019; Ji et al., 2019), although efficacy varies (Becker et al., 2019; Grabau et al., 2021; Liu and Grabau, 2022).

Fluopyram has been tested for PRKN management in Runner-type peanuts with varied results (Grabau et al., 2020, 2024; Hagan et al., 2024). In one large-plot field trial in Florida, fluopyram improved yield in one season but did not manage PRKN abundances and did not affect yield in a second season when PRKN pressure was low (Grabau et al., 2020). In a small plot field study across 7 seasons in Alabama (Hagan et al., 2024), fluopyram did not affect final PRKN soil abundances or root galling but improved peanut yield, which was attributed primarily to the management of early or late leaf spot diseases caused by foliar fungi *Passalora arachidicola* and *Nothopassalora personatum*. In a small plot field study in Florida, fluopyram did not improve yield and generally did not manage PRKN abundances or symptoms, except that treatment with two applications of fluopyram, in-furrow and inseason, reduced root system galling at harvest in one of two seasons (Grabau et al., 2024).

In the same Florida study, resistance (TifNV-High O/L) was more effective than fluopyram or aldicarb in combination with a susceptible Runner-type-type peanut cultivar (Georgia-06G) at managing PRKN abundances and improving yield (Grabau et al., 2024). That is the only peer-reviewed study we are aware of that compares the efficacy of resistance and nematicide application for managing PRKN in peanuts, and additional published research is needed. Furthermore, to our knowledge, fluopyram efficacy for managing PRKN on Virgina-type peanuts has not been reported, although similar results to testing in Runner-type peanuts would be expected.

In addition to target effects on plant-parasitic nematodes, the impact of management practices on non-target organisms is increasingly considered. In particular, non-parasitic, free-living nematodes are a likely off-target casualty of nematode management, particularly nematicide application (Grabau and Chen, 2016; Grabau et al., 2020). Free-living nematodes may feed on microbes or other organisms (Yeates et al., 1993) and are involved in beneficial processes such as nutrient cycling (Melody et al., 2016), decomposition (Holajjer et al., 2016), microbe recolonization (Jiang et al., 2018), and, potentially, management of plant-parasitic nematodes or other pests (Khan and Kim, 2005). While modern nematicides tend to have fewer non-target effects than older carbamate and organophosphate insecticide-nematicides (Desaeger et al., 2020), fluopyram can have deleterious effects on free-living nematodes (Waldo et al., 2019; Grabau et al., 2021). Presumably, this is in part due to the bottom-up effects of fluopyram on the microbial community since fluopyram has activity against fungi (Cordova et al., 2017; Kandel et al., 2019). However, these effects vary by system, and in other situations, fluopyram has not impacted free-living nematodes (Watson and Desaeger, 2019; Grabau et al., 2020).

Resistant cultivars are presumed to have minimal non-target effects because they work by disrupting specialized symbioses between the plant and plant-parasitic nematodes (Bendezu and Starr, 2003). However, regardless of the resistant crop, there is very little field research to verify this assumption. In Minnesota, long-term production of soybeans resistant to *Heterodera glycines* did not affect free-living nematode abundances relative to a susceptible cultivar (Grabau and Chen, 2016). In the Florida Runner-type peanut trial described earlier (Grabau et al., 2024), the PRKN resistant cultivar TifNV-HighO/L consistently reduced final total free-living nematode abundances. Given the negative impact of resistance in that prior peanut study and potential implications for management decisions, further information about the impact of resistant peanut cultivars on free-living nematodes is needed.

In addition to PRKN, several diseases are problematic in Southeast peanut production and may be influenced by cultivar or fluopyram application. Tomato spotted wilt is caused by *Tomato spotted wilt tospovirus* (TSWV) and vectored by thrips feeding on foliage (Culbreath et al., 2008). Stem rot is a soilborne disease caused by *Athelia rolfsii*, while early and late leaf spot are foliar fungal diseases as described earlier (Hagan et al., 2024). Susceptibility to each disease can vary by cultivar (Holbrook et al., 2008; Branch and Brenneman, 2015; Holbrook et al., 2024). Because fluopyram has fungicidal activity, it may also help manage leaf spots and stem rot (Hagan et al., 2024). These diseases were not the target of the study and management practices were designed to control these diseases as well as possible, but they were monitored in this study to help distinguish between any disease and PRKN impacts on yield.

The objectives of this study were to determine the efficacy of resistant Virginia-type peanut cultivars (GA-19HP and TifJumbo) relative to a common susceptible cultivar (Bailey II) with or without in-furrow fluopyram nematicide at (1) managing PRKN and (2) impacts on free-living nematodes in field conditions.

## Materials and Methods

### Site

To investigate these objectives, a field experiment was conducted at the University of Florida Plant Science Research and Education Unit near Citra, Florida (29.404, -82.170) in 2022 and repeated in 2023. The soil type was Gainesville Loamy Sand with 87% sand, 5% silt, 8% clay, 7.65 pH, cation exchange capacity of 7.3 meq, and <1% organic matter. The site has been in continuous peanut, with small grain winter cover crops, for several years. The site was confirmed to have *M. arenaria* based on mitochondrial haplotyping, followed by confirmation using species-specific primers conducted on females extracted from peanut roots in 2021 using the methodology described by Grabau et al. (2020) as developed by Pagan et al. (2015).

### Experimental Design

The experiment was a randomized complete block design with 6 replicates and a single experimental factor: cultivar-nematicide combinations. The treatments were: (1) Bailey II susceptible cultivar, (2) Bailey II with fluopyram, (3) TifJumbo resistant cultivar, and (4) GA-19HP resistant cultivar. Bailey II was distributed by Severn Peanut Company (Severn, NC), and TifJumbo and GA-19HP were distributed by the Georgia Seed Development Commission (Athens, GA). Fluopyram (Velum, Bayer CropScience, St. Louis, MO) was applied in-furrow at 237 g a.i./ha in a total solution rate of 70.3 L/ha from a tractor-mounted planter. The rate used is common for peanuts and near the maximum labeled rate of 250 g a.i./ha. Fluopyram was delivered on top of peanut seeds at planting using a planter-mounted, flat-fan nozzle, and then the furrow was closed by press wheels at the back of the planter. In 2023, the trial was conducted in a different part of the same field as the 2022 trial so there were no residual nematicide treatment effects. Plots were 9.14 m long, and 4 rows (3.66 m) wide, with 91 cm center-to-center row spacing.

### Crop production

Crops were managed conventionally using standard practices for the region. Irrigation was supplied as needed using an overhead center-pivot system. The site was maintained with conventional tillage. Standard fertilizer, herbicide, and fungicide regimes for the region were employed uniformly across the trial (Wright et al., 2021). Aside from fluopyram to selected treatments, no infurrow pesticides were applied to the trial. Peanuts were planted in late April or early May, inverted mechanically in late September, and harvested using a combine when air-dried 2–6 days later (Table 1). Only the central 2 rows of each plot were inverted and harvested with peanuts from each plot collected separately. After inversion, plant roots and pods were checked for galling (5 plants per plot in 2022 and 5 plants per Bailey II plot in 2023), but no galling was observed. Following harvest, peanuts were further dried overnight using a forced-air blower to a target moisture of 10%. Subsequently, peanut yield harvested from 2 rows of each plot was weighed.

**Table 1:** Schedule for data collection and trial establishment. Numbers in parentheses are days after planting.

**Event**	**2022**	**2023**
Preplant soil sampling	28 April (0)	8 May (0)
Planting	28 April (0)	8 May (0)
Stand count 1	10 May (12)	22 May (14)
Vigor rating 1	9 June (42)	19 June (42)
Vigor rating 2	5 July (68)	21 July (74)^a^
Vigor rating 3	2 Aug (95)	28 Aug (112)^b^
Midseason soil sampling	9 June (42)	22 June (42)
Midseason root sampling	9 June (42)	22 June (45)
Harvest soil sampling	6 Sep (129)	6 Sep (121)
Leafspot and stem rot rating	20 Sep (143)	19 Sep (134)
Peanut inverting	20 Sep (143)	19 Sep (134)
Peanut combining	22 Sep (145)	25 Sep (137)

a Tomato spotted wilt was also rated on 21 July 2023.

b Tomato spotted wilt and aboveground stem rot incidence was also rated on 28 August 2023.

### Marginal return on investment

For each plot, the marginal return on investment was calculated based on crop value and treatment cost as described previously for peanut production (Grabau et al., 2024). Briefly, the net return for each plot (*n*) was calculated as *n* = *r* – *c*, with *r* revenue per ha and *c* treatment cost per ha. Revenue was the estimated plot yield multiplied by the estimated peanut price of $0.479/kg (US dollars). Seed cost was estimated to be the same for all cultivars, so fluopyram price was the only cost included in the analysis and was based on local estimates. The mean net return for Bailey II plots without fluopyram (*b*) was calculated. Finally, each plot’s marginal return (m) was calculated as *m* = *n* – b.

### Midseason PRKN quantification from roots

At 6 weeks after planting (Table 1), PRKN populations from roots were quantified. Four peanut plants from each plot were dug from separate locations in the outer plot rows to preserve the central rows from which yield was quantified. Roots were protected from drying and heat by storing them in soil and removing them from sunshine until they were transported to a cold storage room (10 °C) and extracted the following day. Roots were washed gently to remove excess soil and placed on paper towels to dry extra water. Plants were cut at the soil line and fresh shoot and root biomass were measured separately. Roots were also checked for galling, but a near-zero level was detected, so it was not quantified. Then, PRKN were extracted from roots by bleach extraction (Hussey and Barker, 1973) using a VWR 3500 STD orbital shaker (VWR International, PA, USA) as described by Sandoval-Ruiz and Grabau (2023). This extraction yielded primarily PRKN eggs, but any PRKN J2 or males were also counted.

### Soil nematode quantification

Plant-parasitic nematodes and total free-living nematodes were quantified from the soil at planting, 6 weeks after planting, and around harvest (Table 1) each year. Twelve soil cores 25 cm deep were collected from each plot using an Oakfield tube with a 2 cm diameter. At midseason and harvest, cores were collected near plant stems in the central 2 rows of each plot, intersecting with the root system. Samples collected at planting were collected from approximately where the central 2 rows would be located. Nematodes were extracted from 100 cm^3^ soil using the sucrose-centrifugation method (Jenkins, 1964). By microscope (Zeiss Primovert, Carl Zeiss AG, NY, USA), plant-parasitic nematodes were identified based on morphology, typically at 100x magnification. In addition to PRKN, *Mesocriconema* spp. (ring nematode) were consistently present at the site and were included in the analysis. Total free-living nematode abundances were also quantified microscopically, but no taxonomic-level identification was done.

### Peanut growth and disease assessment

Plant stand was assessed approximately 2 weeks after planting (Table 1) by counting plants from a 3.05-m-section in each of the central 2 rows. Plant vigor was rated visually at approximately 6, 10, and 13–15 weeks after planting (Table 1) on a 0–100 scale with 100 most vigorous. Plants were assessed primarily based on canopy size, with a rating of 75 considered a commercial average for that time point, 50 well below average, 25 severely compromised, 0 dead, and 100 extraordinary. At harvest, the foliage was rated for the severity of leaf spot lesions and defoliation using the Florida 1–10 scale (Chiteka et al., 1988), with 10 being the most severe. Just after inversion, the two inverted rows were rated for stem rot hits, based on the presence of blackened stems and crowns, common symptoms of stem rot (Rodriguez-Kabana et al., 1975). As described by Rodriguez-Kabana et al. (1975), each 30.5 cm section of a row with one or more symptomatic plants was considered a hit. Similarly, in 2023, the incidence of Tomato Spotted Wilt symptoms was observed and rated around 10 and 15 weeks after planting (Table 1) using a previously-developed method (Culbreath et al., 1997). Puckered leaves with ringspots were a defining symptom of Tomato Spotted Wilt along with chlorotic, stunted plants. Tomato spotted wilt hits were quantified as described for stem rot. Rating disease incidence as hits in row sections rather than on individual plants is standard in peanuts because plants intertwine, making it difficult to distinguish individual plants (Culbreath et al., 1997).

### Statistical analysis

Each variable was analyzed separately for each trial and sampling date using one-way ANOVA because responses of key variables, namely yield, varied by year. Before completing ANOVA, response variables were transformed, if needed, to meet assumptions of homogeneity of variance using Levene’s test (Levene, 1960) and normality of residuals based on graphing (Cook and Weisburg, 1999). Specifically, PRKN soil abundances at harvest in 2023, PRKN abundances from roots at midseason in 2022 and 2023, and free-living nematode soil abundances at midseason in 2022 and 2023 were transformed by square roots. None of the other variables in the study were transformed. Treatment effects were considered significant at α*=*0.05 in ANOVA. Treatment means were separated by Fisher’s protected LSD (α=0.05) if main effects were significant in ANOVA. Analyses were conducted in R statistical software (version 3.4.4, The R Foundation for Statistical Computing, Vienna, Austria).

## Results

### Peanut root-knot nematode and ring nematode abundances

**Figure 1:**
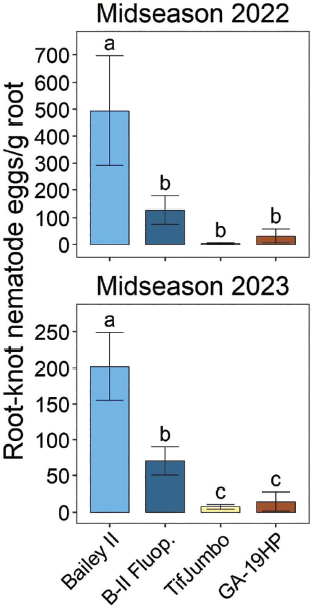
Peanut root-knot nematode egg abundances from peanut roots at midseason (6 weeks after planting) in 2022 and 2023 as affected by cultivars and nematicide treatment. “B-II Fluop.” indicates Bailey II cultivar with infurrow fluopyram application. Other treatment names indicate cultivars without nematicide application. Within each subfigure, means with different letters are significantly different (Fisher’s protected LSD, α = 0.05).

In both years, resistant cultivars or fluopyram application significantly reduced midseason PRKN abundances from peanut roots (Fig. 1). In 2023, either resistant cultivar also significantly reduced midseason PRKN abundances from roots relative to Bailey II with fluopyram, but there were no differences between these treatments in 2022 (Fig. 1). In both years, PRKN was nearly absent in roots of resistant cultivars with reductions of 92–98% relative to Bailey II without fluopyram. Fluopyram reduced PRKN from roots by 65% in 2022 and 74% in 2023.

**Figure 2:**
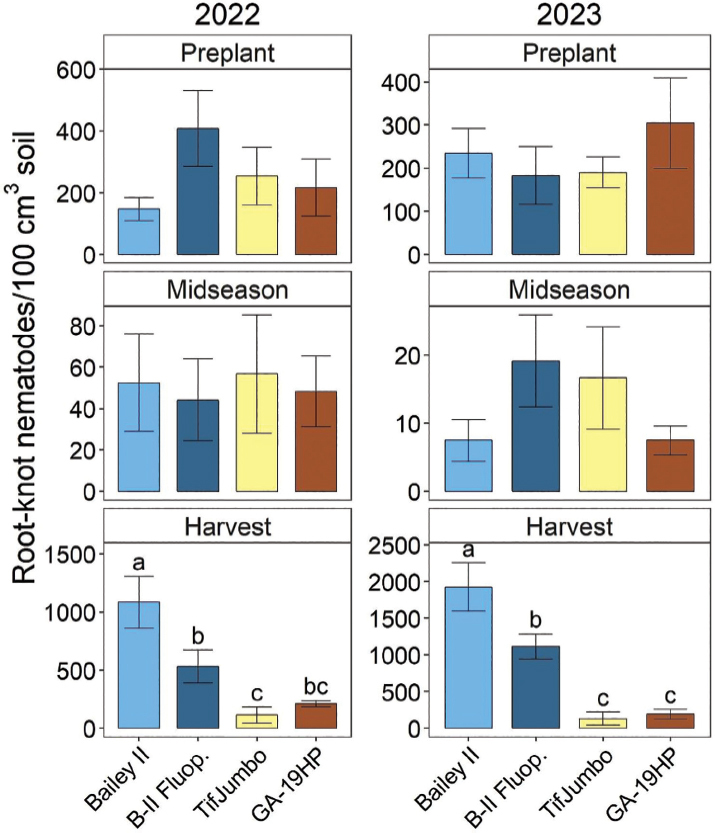
Peanut root-knot nematode soil abundances before planting, at midseason (6 weeks after planting), and at harvest in 2022 and 2023 as affected by cultivars and nematicide treatment. “B-II Fluop.” indicates Bailey II cultivar with in-furrow fluopyram application. Other treatment names indicate cultivars without nematicide application. Within each subfigure, means with different letters are significantly different (Fisher’s protected LSD, α = 0.05).

**Figure 3:**
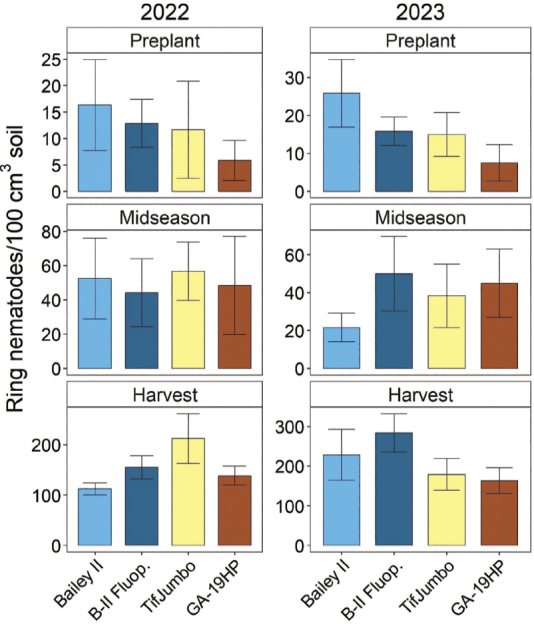
Ring nematode soil abundances before planting, at midseason (6 weeks after planting), and at harvest in 2022 and 2023 as affected by cultivars and nematicide treatment. “B-II Fluop.” indicates Bailey II cultivar with in-furrow fluopyram application. Other treatment names indicate cultivars without nematicide application. Within each subfigure, means with different letters are significantly different (Fisher’s protected LSD, α = 0.05).

Peanut root-knot nematode soil abundances were affected by treatments only at harvest (Fig. 2). Harvest PRKN soil abundances generally decreased significantly stepwise with Bailey II greatest, followed by Bailey II with fluopyram, and resistant cultivars least, except that GA-19HP was not significantly different from the fluopyram treatment in 2022 (Fig. 2). Fluopyram reduced the final PRKN abundances 42% in 2022 and 51% in 2023. Resistant cultivars reduced final PRKN abundances by 90–93% in 2022 and 81–89% in 2023. Treatments did not significantly affect ring nematode soil abundances in any season (Fig. 3).

### Peanut yield and marginal return on investment

**Figure 4:**
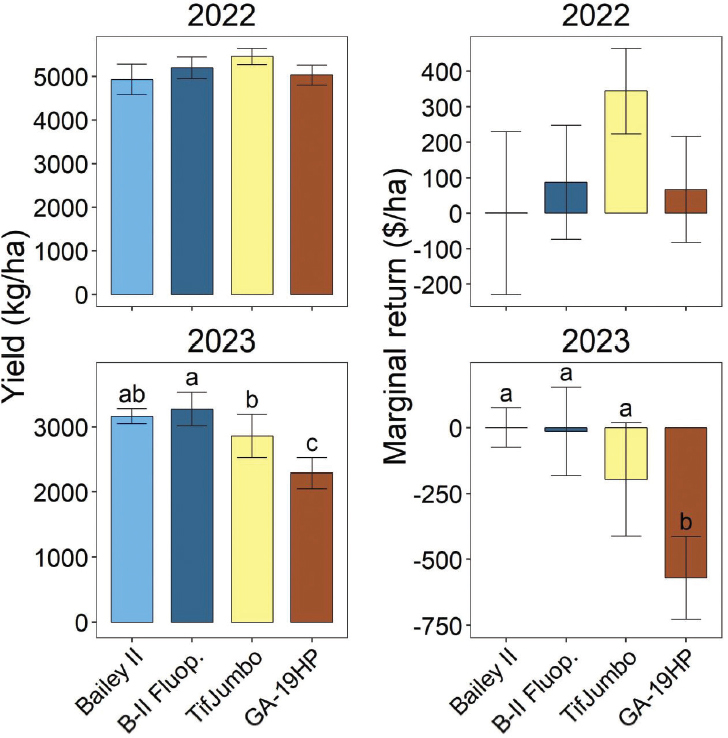
Peanut yield and marginal return on investment in 2022 and 2023 as affected by cultivars and nematicide treatment. “B-II Fluop.” indicates Bailey II cultivar with in-furrow fluopyram application. Other treatment names indicate cultivars without nematicide application. Marginal return (in US Dollars) is relative to mean income (crop value minus product cost only) for Bailey II. Within each subfigure, means with different letters differ significantly (Fisher’s protected LSD, α = 0.05).

Peanut yield did not vary significantly by cultivar or nematicide in 2022 (Fig. 4). In 2023, the yield was less for the resistant cultivars than Bailey II with fluopyram and also less for GA-19HP than Bailey II without fluopyram. Marginal return on investment was not significantly affected by treatments in 2022. Still, TifJumbo had a marginal return of $344/ha relative to Bailey II without nematicide, which would be meaningful to growers (Fig. 4). In contrast, marginal return was significantly decreased by GA-19HP in 2023 (-$570/ha), relative to all other treatments, and TifJumbo also had a numerically negative (-$197/ha) marginal return.

### Vegetative peanut growth

Plant stand was influenced by treatment in both years but had varying trends. In 2022, Bailey II without fluopyram had a greater stand than TifJumbo or GA-19HP (Table 2). In contrast, in 2023, either resistant cultivar had a greater stand than Bailey II treatments. Shoot weight at midseason was greater for Bailey II, with or without fluopyram, in 2022 but not significantly affected by treatments in 2023 (Table 2). Root weight at midseason was not significantly affected by treatments in either year (Table 2). In both years, plant vigor was generally greater for Bailey II treatments than either resistant cultivar across assessment dates (Table 2). At the final dates, vigor was assessed (95 days in 2022 and 112 days after planting in 2023), and plant vigor was also less for GA-19HP than TifJumbo (Table 2).

**Table 2:** Cultivar and nematicide effects on agronomic measurements in 2022 and 2023 trials. Numbers in parentheses are days after planting.

Treatment	----------------------------------------2022---------------------------------------							
	Stand (12)	Root weight (42)^b^	Shoot weight (42)^b^	Vigor (42)	Vigor (68)	Vigor (95)	Stem rot (143)^c^	Leafspot (143)^d^
Bailey II	3.0 a	4.6	42 a	87 a	89 a	85 a	1	5.8 a
Bailey II + Fluopyram	2.8 ab	3.6	34 a	88 ab	95 a	85 a	1	6.3 a
TifJumbo	2.5 b	3.9	33 ab	67 c	63 b	74 b	1	4.7 b
Georgia-19HP	2.5 b	3.5	21 b	77 bc	50 b	59 c	2	3.5 c

**Treatment**	**----------------------------------------2023---------------------------------------**							
	**Stand (14)**	**Root weight (42)**	**Shoot weight (42)**	**Vigor (42)**	**Vigor (74)**	**Vigor (112)**	**Stem rot (134)**	**Leafspot (134)**
Bailey II	2.1 b	5.3	76	53 b	68 ab	70 ab	1 b	0.0 b
Bailey II + Fluopyram	1.9 b	5.8	81	60 a	72 a	73 a	1 b	0.7 ab
TifJumbo	2.6 a	5.6	78	56 ab	63 bc	68 b	1 b	0.0 b
Georgia-19HP	2.7 a	5.3	60	55 ab	59 c	62 c	3 a	1.8 a

a Different letters for the same variable and year indicate significantly different means based on Fisher’s protected LSD at α = 0.05.

b Root and shoot weights are in grams per plant

c Stem rot hits per meter of row. A hit is a 30.5 cm section of row with symptomatic plants

d Leafspot is leafspot severity rating on a Florida 1–10 scale, where 10 is the most severe (Chiteka et al., 1988)

### Disease ratings

There were no significant differences in stem rot loci at harvest in 2022, but there were more loci in GA-19HP than in other cultivars in 2023 (Table 2). In 2022, the leaf spot rating at harvest was greater for Bailey II than for the resistant cultivars (Table 2). In 2023, the leafspot rating was higher for GA-19HP than the other cultivars, but the severity was low overall (Table 2). In 2023, the percent of discolored pods at harvest was significantly (Fisher’s protected LSD, *P* < 0.05) greater for GA-19HP (23%) than other cultivars (15%, data not shown). In 2023, tomato spotted wilt incidence was not significantly affected by treatments, with an average of 3 and 5 hits/ plot at 74 and 112 days after planting, respectively (data not shown).

### Free-living nematodes

**Figure 5:**
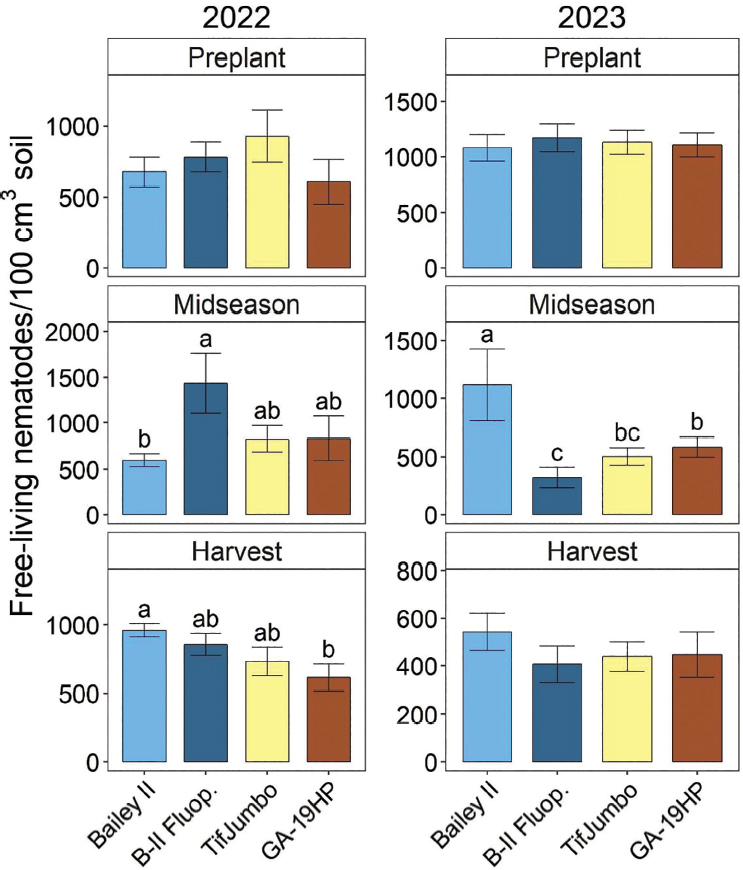
Total free-living nematode soil abundances before planting, at midseason (6 weeks after planting), and at harvest in 2022 and 2023 as affected by cultivars and nematicide treatment. “B-II Fluop.” indicates Bailey II cultivar with in-furrow fluopyram application. Other treatment names indicate cultivars without nematicide application. Within each subfigure, means with different letters differ significantly (Fisher’s protected LSD, α = 0.05).

Free-living nematode soil abundances were significantly affected by treatments at midseason in both years and harvest in 2022, but not at planting either year or harvest in 2023 (Fig. 5). In midseason 2022, free-living nematode soil abundances were significantly greater for Bailey II with fluopyram than without and intermediate for the resistant cultivars (Fig. 5). In contrast, at midseason in 2023, either fluopyram or resistant cultivars reduced free-living nematode abundances relative to Bailey II without fluopyram. Fluopyram also reduced free-living nematode abundances relative to GA-19HP at midseason in 2023. At harvest in 2022, free-living nematode abundances were significantly less for GA-19HP than Bailey II, with the fluopyram treatment and TifJumbo intermediate.

## Discussion

From this study, resistance and fluopyram nematicide application were both effective tools for managing PRKN abundances and infection in peanuts. Resistance was slightly more effective than fluopyram, especially for controlling end-ofseason abundances, which may provide PRKN management benefits for the following crop. However, PRKN population control did not translate to improved peanut yield. Based on observations from this study and past studies in this field, we hypothesize that this was because the PRKN isolate in this field is avirulent or has reduced virulence (i.e., damage potential) on peanuts. It follows that inconsistent yield by GA-19HP and TifJumbo was due to cultivar yield potential and possibly other diseases rather than any effects from PRKN.

As described in the results, there was no detectable damage (galling, chlorosis, reduced yield, or compromised canopy vigor) to the susceptible peanut cultivar from PRKN in this study. While there were differences in plant vigor among cultivars, the susceptible Bailey II was more vigorous than the resistant cultivars, a difference likely due to cultivar phenotype rather than PRKN management, as Bailey II is anecdotally known for vigorous vine growth. The PRKN isolate in this field was infecting and reproducing on peanuts based on abundant PRKN eggs extracted from Bailey II roots at midseason and increasing PRKN soil abundances during the year. Based on the root and soil counts, the level of PRKN infestation in the field was severe. Typically, treatments that effectively manage PRKN under severe infestation produce a dramatic increase in yield and alleviation of chlorosis and other PRKN symptoms, whether nematicides (Kinloch and Dickson, 1991) or resistant Runner-type peanut cultivars (Holbrook et al., 2008, 2017; Grabau et al., 2024). Anecdotally, the lack of damage to peanuts in this study does not seem to result from broad host tolerance by Virginia-type peanuts because damage from PRKN has been observed by the authors in local Virgina-type peanut fields.

Again, the lack of symptoms on peanuts suggests that the PRKN isolate at the study field site was avirulent or had reduced virulence. Variation in virulence among isolates of a single *Meloidogyne* species is well-established (Rutter et al., 2021; Ploeg et al., 2023), including for PRKN (Noe, 1992). However, the extreme de-coupling of PRKN fecundity and damage in this study—high fecundity, no detectable damage—is atypical as when avirulent isolates cause little or no crop damage, it is typically because they do not reproduce well on the host crop (Noe, 1992; Rutter et al., 2021; Ploeg et al., 2023). Other hypotheses on the host or environment side are feasible and further research would be needed to support the avirulence hypothesis further. Additionally, further research to determine the frequency of PRKN reproduction without symptoms on peanuts would be useful from a theoretical and practical perspective.

Cultivar selection is an important choice for peanut producers seeking to maximize yield and profitability and this study guided this process. In this study, TifJumbo and GA-19HP had less consistent agronomic performance and return on investment than Bailey II, especially when combined with fluopyram. Based on these results, if a grower was unsure about the level of PRKN pressure in their field, selecting Bailey II and supplementing with fluopyram application may be the safest choice among cultivars tested in this study since it performed well under effectively low PRKN pressure in this study and provided good control of PRKN populations, so would presumably perform well under high PRKN pressure.

However, these study results contrast prior multiyear, multi-location studies that were not selected for high PRKN pressure (Branch and Brenneman, 2020; Holbrook et al., 2024). Across 30 tests for GA-19HP registration data, TifJumbo and GA-19HP had similar or better yields than Bailey, which is nearisogenic to Bailey II (Branch and Brenneman, 2020). TifJumbo and Ga-19HP outyielded Bailey II by 20% and 11%, respectively, across 3 Georgia locations in 2019 tests (Holbrook et al., 2024). In 2023, stem rot and leafspot diseases may have contributed to poor agronomic performance by GA-19HP as the incidence of disease symptoms was greater for GA-19HP than other cultivars that year. Ring nematodes are unlikely to have affected peanut yield as cultivars and nematicides did not affect ring nematode abundances, and this nematode is not known to have a substantial impact on peanut yield. Other explanations, such as environmental conditions unfavorable for certain cultivars, are also possible. Further testing under more substantial PRKN pressure is needed since resistance will most likely be deployed and effective under those conditions.

The high level of resistance exhibited by Virginiatype resistant cultivars in this study is consistent with prior research on these Virginia-type cultivars (Branch and Brenneman, 2020; Holbrook et al., 2024) as well as resistant Runner-type peanuts (Holbrook et al., 2008, 2017; Grabau et al., 2024). In this study, the good control of PRKN abundances at harvest suggests that resistant cultivars will not only reduce PRKN in that peanut crop, but it may also have carryover benefits for the next season, which is also similar to work in resistant Runner-type peanut (Branch and Brenneman, 2023; Grabau et al., 2024). The lack of impacts on midseason soil abundances reflects the variability of soil PRKN populations at that time (Grabau et al., 2020, 2024). This suggests that PRKN populations from roots are a more useful tool than PRKN soil abundances at midseason for evaluating PRKN management treatments.

Fluopyram was useful for managing PRKN in this study, which is in contrast to prior Runner-type peanut studies where fluopyram generally did not manage abundances or galling and had inconsistent impacts on yield (Grabau et al., 2020, 2024; Hagan et al., 2024). Environmental factors are the most likely explanation for the improved fluopyram performance in this study, particularly soil type. The soil in this study was slightly heavier than in past studies (Grabau et al., 2020, 2024; Hagan et al., 2024). Fluopyram is poorly mobile and binds to clay particles (Faske and Brown, 2019), so it is possible that greater clay content increases the residual activity of fluopyram in this field. Comparisons of fluopyram performance under varying soil types via controlled experiments or metanalysis would be useful to validate this hypothesis. Better understanding fluopyram performance across soil types may guide growers in selecting the most effective nematicide or management practice for their soil type. This would be broadly useful across cropping systems as fluopyram performance at managing root-knot nematodes has been inconsistent across studies in different systems and locations (Becker et al., 2019; Ji et al., 2019; Dahlin et al., 2019; Grabau et al., 2024). It is also possible that fluopyram performance in this study was improved because the Virginia-type peanut cultivar Bailey II is more susceptible to PRKN (i.e. supports more reproduction) than susceptible Runner-type peanut cultivars used in past studies, namely Georgia-06G (Grabau et al., 2020, 2024; Hagan et al., 2024). This could magnify treatment differences. Further assessment of Virginia-type peanut cultivars’ susceptibility and tolerance to PRKN would be useful for guiding field management since there is little work on this topic.

Resistant cultivars and fluopyram had inconsistent impacts on free-living nematodes. In row crops, where fluopyram application is concentrated in the planting furrow, fluopyram has typically had minimal non-target effects on free-living nematodes (Grabau et al., 2020, 2024). In contrast, fluopyram has had more negative impacts in cropping systems where application rates and coverage areas are greater (Waldo et al., 2019; Grabau et al., 2021). Although inconsistent, resistant cultivars negatively impacted free-living nematodes, reducing abundances in both years (harvest 2022 and midseason 2023). These results, combined with the negative impacts of resistant Runner-type peanut (TifNV-HighO/L) in a prior study (Grabau et al., 2024), make investigating the impact of resistant peanut cultivars on free-living nematodes an area of interest. Typically, resistance is considered a more environmentally friendly management option than nematicide application, so negative effects on non-target organisms would shift this perception.

It is unlikely that resistant cultivars have a direct negative impact on free-living nematodes since resistance is conferred by disrupting the specialized plant-nematode interaction that occurs within the plant root system (Simpson and Starr, 2001; Bendezu and Starr, 2003) and free-living nematodes do not feed on root tissue. A previously proposed (Grabau et al., 2024) mechanism for this observation is that susceptible cultivars release a greater amount or variety of root exudates than resistant cultivars under PRKN infection, which could have a bottom-up effect on the microbial community and, in turn, free-living nematodes. Prior research supports that PRKN-resistant cultivars have different root gene expression profiles than susceptible cultivars (Tirumalaraju et al., 2011). However, it is unknown if those differences affect root exudate profiles or the microbiome. There are examples of other pathogens altering root exudate profiles or the microbiome. Root exudate profiles varied for peanut cultivars with varying resistance levels to *Fusarium* pathogens (Li et al., 2013). The rhizosphere and endosphere microbiome varied for cotton cultivars with different levels of *Verticillium dahlia* resistance (Wei et al., 2019). Further research would be needed to support this hypothesis of a bottom-up mechanism for the negative impacts of PRKN resistance on free-living nematodes. Additionally, further work assessing which taxonomic and trophic groups of free-living nematodes are affected by resistant peanut cultivars is needed as this has implications for the soil community and has not been investigated (Grabau et al., 2024).

In summary, this study advanced the understanding of PRKN management by resistance and nematicide application in Virginia-type peanuts. Resistance was a highly effective tool for reducing PRKN infection and final PRKN soil abundances, which can help manage subsequent crops. Similarly, fluopyram application to a susceptible cultivar (Bailey II) was also effective, although at a slightly reduced level compared with resistance. Management of PRKN did not translate to improved yield, which may be the result of a PRKN isolate with reduced virulence or environmental factors of the site. The inconsistent yield of resistant cultivars, particularly GA-19HP, suggests yield potential is lower or less consistent than the susceptible cultivar Bailey II when PRKN pressure is low. Under unknown or low PRKN pressure, Bailey II with fluopyram would be the safest choice for growers based on this study. Non-target impacts on free-living nematodes were inconsistent, although negative impacts of resistant cultivars warrant further attention. Future research is needed to evaluate Virginia-type peanut cultivar performance and fluopyram under severe PRKN pressure.
